# Comparison of Traditional Bougie Versus Kiwi-D Grip Bougie Technique During Mechanical Chest Compressions: A Randomized Crossover Manikin Trial

**DOI:** 10.7759/cureus.77280

**Published:** 2025-01-11

**Authors:** Kaitlin Hunt, Shani B Italiya, Craig Pedersen, K Tom Xu, Colin Kenny, Peter Richman

**Affiliations:** 1 Emergency Medicine, CHRISTUS Spohn Hospital Corpus Christi - Shoreline, Texas A&amp;M (Agricultural and Mechanical) University College of Medicine, Corpus Christi, USA; 2 Surgery, Texas Tech University Health Sciences Center (TTUHSC) School of Medicine, Lubbock, USA; 3 Family and Community Medicine, Texas Tech University Health Sciences Center (TTUHSC) School of Medicine, Lubbock, USA; 4 Emergency Medicine, Baylor College of Medicine at Christus Children’s, San Antonio, USA

**Keywords:** bougie, chest compressions, kiwi-d grip bougie, manikin simulation, resident training

## Abstract

Background: Investigators have reported bougie use improves first-pass intubation success rates when compared to the endotracheal (ET) tube/stylet technique. We aimed to assess the difference in time to intubation and operator confidence between the Kiwi-D grip bougie and traditional bougie technique during simulated mechanical cardiopulmonary resuscitation (mCPR).

Methods: This study was a prospective, randomized comparative study at a simulation center. Consenting emergency physicians were surveyed about intubation experience, and provided structured practice for techniques. Subjects performed direct laryngoscopy (DL) using a Mac 4 blade (Karl Storz SE & Co. KG, Tuttlingen, Germany) on an adult manikin with a moderately difficult airway, during simulated mCPR (LUCAS 3.0, Stryker Corporation, Kalamazoo, MI, USA) at 100 compressions/min. Each subject was intubated using Kiwi-D and traditional bougie techniques, respectively, in a randomized order. A study author measured intubation time (blade pick up until cuff inflation) and assessed intubation success. Subjects rated intubation success confidence on a five-point scale and provided Cormack/Lehane grade. Categorical data was analyzed by chi-square and continuous data by t-tests for bivariate analyses. Multivariate linear regression was performed for intubation time. Non-parametric Wilcoxon signed-rank test was performed for the ordinal categorical variables.

Results: There were 31 subjects; 87% with one to five years of experience, 52% preferred DL during CPR, 71% preferred the traditional no-preload bougie technique, and 48% had utilized a bougie >10 times. Subjects had first-pass intubation success for all but one attempt with both modalities (NS). For Kiwi-D versus traditional bougie, 48% of subjects rated a higher level of confidence for successful intubation (p=0.01), and 29% (p=0.1) reported improved glottic view. Mean time to intubation was similar for Kiwi-D versus traditional (20.6+/-9 versus 25.3+/-17s; p=0.06). The following subject characteristics were not associated with improved intubation time for Kiwi-D: 6+ years of experience (p=0.6), >10 prior intubations with a bougie (p=0.6), preloading bougie preference (p=0.4), and DL preference (p=0.4). Multivariate linear regression did not identify subject variables that were significantly associated with Kiwi-D use for improved intubation time with Kiwi-D.

Conclusion: Subjects in our study group did not have significant differences in time to intubation using Kiwi-D versus traditional bougie during simulated mCPR.

## Introduction

In the past two decades, there has been evidence that minimizing stoppage of chest compressions during cardiopulmonary resuscitation (CPR) improves cerebral and cardiovascular perfusion, thereby, increasing the odds of meaningful neurological survival for patients with cardiac arrest [1]. Current advanced cardiac life support (ACLS) guidelines recommend delay of endotracheal intubation to prioritize tissue perfusion [2,3]. Meanwhile, physicians who attempt to intubate during chest compressions versus holding chest compressions may potentially encounter more difficulty as there is an increased likelihood of airway obstruction and emesis in the oropharynx following bag-mask ventilation [4,5].

With such challenges in mind, we have performed a series of studies evaluating techniques for intubation during non-interrupted mechanical CPR (mCPR) [6,7]. One potential adjunct to assist clinicians during intubation of patients undergoing chest compressions is a bougie. Recently, investigators have shown the potential advantage of utilizing a bougie to improve first success rates in other difficult airway scenarios [8]. Further, authors have suggested that the bougie technique itself might be improved by utilizing a bougie curled upon itself that is inserted through the endotracheal tube prior to initiating intubation (Kiwi-D technique) [9]. Intuitively, one would assume that the need to pause during intubation and load an endotracheal tube onto a bougie would render the traditional method significantly slower. As there is a paucity of data comparing these techniques, we conducted a pilot study to compare the Kiwi-D technique to the traditional bougie method under simulated mCPR conditions.

This article was previously presented as an abstract at the Annual Meeting for the Society of Academic Emergency Medicine, Austin, Texas, May 17, 2023.

## Materials and methods

Study design

This study was a prospective, randomized, comparative study to evaluate clinicians' ability to perform airway adjunctive maneuvers under simulated conditions.

Setting

The study was conducted at a simulation center based within CHRISTUS Spohn-Shoreline Hospital in Corpus Christi, Texas (USA). The facility serves as the primary teaching site for an emergency medicine residency. The study was approved by the CHRISTUS Health Institutional Review Board (Approval No. 2022-188, dated June 15, 2022) prior to the initiation of data collection.

Participants

Subjects were either residents or faculty of a single residency program in emergency medicine.

Study protocol

Consenting emergency physicians were surveyed to collect demographic characteristics and to assess prior intubation experience. The lead study author, subsequently, provided a brief structured training, and each participant was allowed two practice attempts each with traditional and Kiwi-D bougie simulated intubation, respectively. The traditional bougie technique involves placing the bougie into the trachea and then intubating with the endotracheal tube over the bougie. The Kiwi-D technique involves placing the bougie through the endotracheal tube but with this method, the majority of the bougie is placed beyond the distal aspect of the endotracheal tube with a small proximal tip of the bougie just outside the proximal end of the endotracheal tube. The endotracheal tube is then bent in a loop and the proximal end of the bougie is stuck through the Murphy's eye near the distal tip of the endotracheal tube. The practitioner then holds the endotracheal tube just above the balloon with the more proximal portion of the looped endotracheal tube anterior to the hand and guides the bougie into the airway.

Subjects performed direct laryngoscopy (DL) using a Mac 4 blade (Karl Storz SE & Co. KG, Tuttlingen, Germany) on a moderately difficult airway, adult manikin during simulated mCPR (LUCAS 3.0, Stryker Corporation, Kalamazoo, MI, USA) at 100 compressions/min. We chose a moderately difficult airway consistent with prior published methods [10,11]. The endotracheal (ET) tube size was 7.5 mm. Each subject was intubated using Kiwi-D and traditional bougie techniques once, respectively, in a randomized, crossover manner. The randomization was performed by a coin flip for each subject to determine the order of technique. A study author measured intubation time (blade pick up until cuff inflation) and assessed intubation success. Subjects rated intubation success confidence on a five-point scale and provided Cormack/Lehane grade for the airway visualization.

Statistical analysis

Data was entered into Excel 2021 for Windows (Microsoft Corp., Redmond, WA, USA), and imported into STATA 18 statistical software (StataCorp LLC, College Station, TX, US) for analysis. Categorical data are presented as frequency of occurrence and were analyzed by chi-square. Continuous data are presented as means+/-SD and were analyzed by t-tests. Subsequently, multivariate linear regression was performed for intubation time. A non-parametric Wilcoxon signed-rank test was performed for the ordinal categorical variables.

## Results

There were 31 subjects who participated. Within the study group, 87% had one to five years of clinical experience, 52% preferred DL during CPR, 71% preferred the traditional no-preload bougie technique, and 48% had utilized a bougie >10 times (Table 1).

**Table 1:** Subject experience and intubation preferences.

N = 31 subjects	%
1-5 years experience	87%
Direct laryngoscopy as the preferred intubation method during cardiopulmonary resuscitation (CPR)	52%
Preference for no preload bougie technique	71%
48% with prior bougie experience >10 previous intubations	48%

Subjects had first-pass intubation success for all but one attempt each for the two techniques, respectively (NS). For Kiwi-D versus traditional bougie, 48% of subjects rated a higher level of confidence for successful intubation (p=0.01), and 29% (p=0.1) reported improved glottic view. There was a trend that participants had faster intubation times for Kiwi-D versus traditional technique but this did not reach statistical significance (20.6+/-9 versus 25.3+/-17s; p=0.06). The following subject characteristics were not associated with improved intubation time for Kiwi-D: 6+ years of experience (p=0.6), >10 prior intubations with a bougie (p=0.6), preloading bougie preference (p= 0.4), and DL preference (p=0.4). Multivariate linear regression did not identify subject variables that were significantly associated with Kiwi-D use for improved intubation time with Kiwi-D (Table 2).

**Table 2:** Multivariate logistic regression analysis (association with time to intubation).

Subject characteristic	p-value
Intubation experience 6+ years	0.4
Prior bougie use >10 times	0.6
Preference for traditional bougie intubation	0.6
Preference for video laryngoscopy intubation	0.4

## Discussion

In previous investigations at our center, we evaluated several techniques to improve intubation success rates while continuing chest compressions during simulated cardiac arrest scenarios [6,7]. Munion et al. evaluated emergency physician performance of intubation under simulated device-controlled mCPR versus traditional human compressions [6]. Though the trend did not reach statistical significance within a small emergency physician study group, the authors found shorter mean intubation times during mCPR versus manual chest compressions (15.1+/-12 versus 20.5+/-19 sec; p=0.06). 

Roberts et al. conducted a randomized crossover study that compared direct versus video laryngoscopy on an adult manikin with a moderately difficult airway during simulated mCPR [7]. Within this study group of emergency physicians, subjects had significantly shorter mean time to successful intubations for direct laryngoscopy than for video laryngoscopy (-6.8 seconds; p=0.02) though this difference is unlikely to be clinically meaningful. However, clinicians reported “high confidence” for successful intubation more frequently with the use of video laryngoscopy (direct 60% versus video 77%; p=0.2).

Intubations in the setting of CPR and other emergencies are commonly difficult due to ongoing chest compressions, emesis, other obstructing factors in the airway, and/or cervical immobilization [8]. Clinicians in such scenarios may utilize a bougie to overcome situations where visualization might be otherwise limited as the ribbed nature of the airway and tactile stimulus of the bougie facilitates intubation in such situations. Driver et al. conducted a randomized trial involving 757 adult patients with at least one difficult airway characteristic who either were intubated with the guidance of a bougie or with a more traditional ET tube plus stylet method. They found that the first pass success rate was significantly higher in the bougie group {98% versus 87%; absolute difference, 11% (95% CI, 7% to 14%)} [7].

Although the Kiwi-grip technique has been qualitatively described within discussions of various airway techniques, there is a paucity of clinical research evaluating the effectiveness of this technique versus the traditional bougie method [8]. We believe our current study is novel for evaluating the use of the Kiwi-D technique under simulated mCPR conditions. We found a trend that mean time to intubation was faster for the Kiwi-D versus the traditional bougie technique though this difference was not statistically significant (20.6+/-9 versus 25.3+/-17s; p=0.06). It is not clear that such a time difference is clinically meaningful during CPR, though it might be in other clinical circumstances such as a rapidly closing airway. We note that the observed intubation times with a Kiwi preloaded technique for our study group are similar to those observed in two prior small simulation studies for moderately difficult airways in the anesthesia literature (16.5s traditional versus 16.5s preloaded) and an emergency medicine journal (29.7s versus 29.4s) [10,11].

Our pilot study is limited by a small sample size, and, thus, larger studies would be needed to identify whether the trend we observed for faster intubation time for the Kiwi-D versus the traditional bougie method would reach statistical significance. While the time to intubation was similar, the Kiwi-D technique was associated with higher operator confidence, which could have clinical implications in settings with high-stress and difficult airways. Future investigators should evaluate the use of this technique with clinical trials in actual resuscitation settings and with a higher proportion of practitioners with more intubation experience.

## Conclusions

Emergency physician subjects in our study group did not have significant differences in time to intubation using Kiwi-D versus traditional bougie during simulated mCPR. Larger studies are warranted to confirm our results. Further, future studies should compare the two techniques in the clinical setting for both medical and trauma-related airway/respiratory emergencies.
